# Topical Neck Cooling Prolongs Survival of Rats with Intra-Abdominal Feculent Sepsis by Activation of the Vagus Nerve

**DOI:** 10.3390/ijms22189828

**Published:** 2021-09-11

**Authors:** Aimee Y. Zhang, Katherine M. Marsh, Radhika Rastogi, Di Wu, Eric J. Charles, Irving L. Kron, Robert G. Sawyer, Zequan Yang

**Affiliations:** 1 Department of Surgery, University of Virginia Health System, Charlottesville, VA 22903, USA; ayz7kr@virginia.edu (A.Y.Z.); kmm9th@virginia.edu (K.M.M.); rr4wd@virginia.edu (R.R.); dw6a@virginia.edu (D.W.); ec4wx@virginia.edu (E.J.C.); ilk@virginia.edu (I.L.K.); 2 Department of Surgery, Western Michigan University Medical School, Kalamazoo, MI 49008, USA; robert.sawyer@med.wmich.edu

**Keywords:** intraabdominal sepsis, shock, topical hypothermia, neck cooling, vagus nerve, inflammatory response

## Abstract

Global hypothermia prolongs survival in rats with intraabdominal feculent sepsis by inhibiting inflammatory responses. We hypothesized that topical neck cooling (TNC) has similar benefits. Septic shock was induced by cecal ligation and incision (CLI) in Sprague Dawley rats. Rats were randomized to sham laparotomy, control with CLI, CLI with TNC, or vagotomy at the gastroesophageal junction before CLI and TNC. Two more groups underwent peritoneal washout with and without TNC two hours after CLI. TNC significantly lowered neck skin temperature (16.7 ± 1.4 vs. 30.5 ± 0.6 °C, *p* < 0.05) while maintaining core body normothermia. TNC rats recovered from anesthesia 70 min earlier than the control (*p* < 0.05). Three hours following CLI, the control and vagotomy with TNC groups had significantly more splenic contraction, fewer circulating leukocytes and higher plasma IL-1β, IL-10 and TNF-α levels than TNC rats (*p* < 0.05). TNC prolonged survival duration after CLI by a median of four hours vs. control (*p* < 0.05), but no benefit was seen if vagotomy preceded TNC. Peritoneal washout alone increased survival by 3 h (9.2 (7.8–10.5) h). Survival duration increased dramatically with TNC preceding washout, to a 56% survival rate (>10 days). TNC significantly prolonged the survival of rats with severe intraabdominal sepsis by inhibiting systemic proinflammatory responses by activating vagal anti-inflammatory pathways.

## 1. Introduction

The current treatment of sepsis consists of early recognition, antibiotic coverage, timely source control and supportive care to preserve organ function, a protocol that has remained largely unchanged for decades with few new therapies incorporated into standard practice [1,2,3]. Definitive and early infectious source control is the key for treatment, specifically in intraabdominal sepsis, which is the second most common cause of sepsis [3,4]. Treatments to prolong the therapeutic window before definitive source control procedures are performed could prevent patients with intraabdominal sepsis from progressing into septic shock and/or multiple organ failure. Therapeutic hypothermia, achieving a core temperature between 32–35 °C, has been recognized as a potential treatment for septic shock [5,6], given its well-established benefits in neuroprotection [7,8,9] and organ preservation following cardiac arrest [10,11,12,13]. It produces multifactorial beneficial effects resulting in an overall protective anti-inflammatory state. We have previously demonstrated in a murine sepsis model that systemic hypothermia significantly reduces pro-inflammatory responses and prolongs survival duration during severe septic shock [14]. This therapy, therefore, presents an attractive approach to inhibit the pathogenic immunological processes responsible for tissue injury and organ failure in sepsis.

Various delivery methods for therapeutic hypothermia have been studied, including both global and topical hypothermia. Thus far, global hypothermia has been the treatment of choice in therapeutic hypothermia studies and applications across multiple disease processes [10,12,15,16,17,18]. However, a recent large-scale clinical trial demonstrated that therapeutic global hypothermia did not provide any mortality benefit in treating septic shock [15]. The potential risks associated with global hypothermia include arrhythmia [19], increased peripheral vascular resistance, hemorrhage [20,21,22], and increased risk of infection [23]. These risks may outweigh its therapeutic benefits and explain the lack of mortality benefit in a clinical setting. Of note, the trial’s inclusion criteria of respiratory failure requiring mechanical ventilation indicates that fulminant sepsis had already ensued in these patients and consequently, may have progressed too far towards multi-organ failure for this therapy to positively intervene. Global hyperthermia is also impractical in pre-hospital settings given its associated side effects, high resource utilization, and demand for critical care. Finally, global hypothermia below 32 °C must be avoided due to increased risk of ventricular arrhythmia. Given these systemic effects, a more regional approach may be beneficial. In the neurologic arena, in place of global hypothermia, topical hypothermia using a helmet or nasal device to cool the brain has been studied for neural protection following stroke, concussion, and cardiac arrest [24,25,26,27]. Recently, we found that topical neck cooling (TNC) to a subcutaneous neck temperature of 14–17 °C while maintaining core body normothermia had similar effects to global hypothermia [14], while potentially avoiding its systemic risks. Furthermore, it is easily applicable, non-invasive, and has a small footprint, all of which would allow for its use in pre-hospital and austere, low-resource settings.

In the current study, we sought to evaluate the effect and mechanisms of TNC in severe intraabdominal sepsis using a rat model. We hypothesized that TNC in severe abdominal sepsis would provide a protective effect and lead to increased survival duration, potentially through a vagal anti-inflammatory pathway.

## 2. Results

### 2.1. Effect of Topical Neck Cooling (TNC) on Hemodynamics in Normal Rats

**Figure 1:**
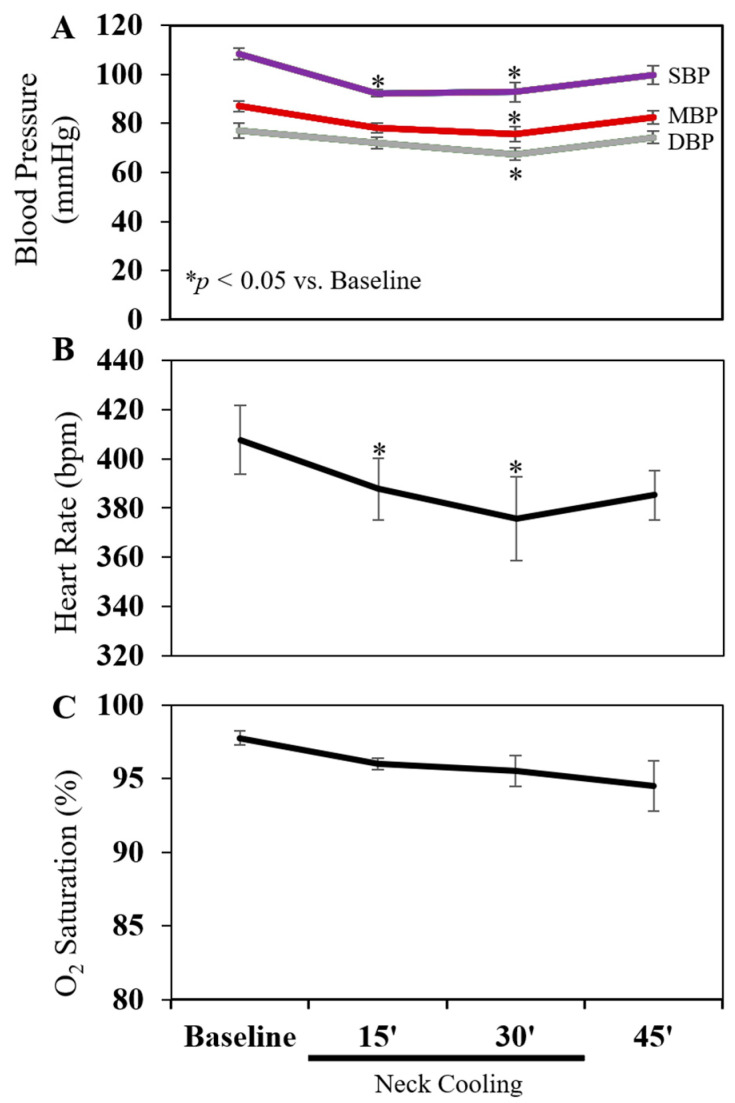
Effect of topical neck cooling on (**A**) blood pressure, (**B**) heart rate and (**C**) oxygen saturation. Four rats were used. SBP: Systolic blood pressure. MBP: Mean blood pressure. DBP: Diastolic blood pressure.

TNC was applied to four normal rats without cecal ligation and incision (CLI) for 30 min. Before applying TNC, the rectal and neck subcutaneous temperatures were 36.6 ± 0.2 °C and 30.6 ± 0.6 °C, respectively. TNC decreased the neck temperature to 14.7 ± 1.1 °C (*p* < 0.05 vs. baseline) without affecting the rectal temperature (36.4 ± 0.2 °C), which was maintained using a heating lamp. TNC caused a significant drop in mean blood pressure from baseline, with a 14% and 17% decrease at 15 and 30 min, respectively, and a significant decrease in pulse of 4% and 9% at those respective timepoints. Blood pressure and pulse quickly recovered to baseline within 15 min of discontinuing the TNC treatment (Figure 1A,B). Oxygen saturation on room air slightly downtrended during TNC, but the decrease did not reach statistical significance (Figure 1C).

### 2.2. Spleen Weight, Circulating WBC and Lung Tissue Myeloperoxidase (MPO) following CLI

**Figure 2:**
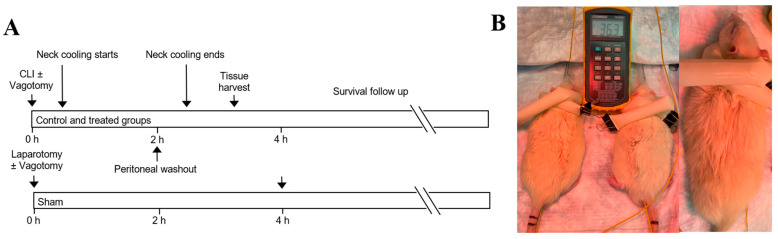
(**A**) Experimental protocol. (**B**) Topical neck cooling with ice-filled Penrose. CLI: cecal ligation and incision.

CLI was used to induce intraabdominal feculent sepsis. Rats were randomized into a sham laparotomy group, a CLI control group, or CLI followed by TNC with or without vagotomy at the gastroesophageal junction (GEJ) (Figure 2A). In TNC groups, the neck was wrapped with an ice-filled 1-inch Penrose drain 30 min after CLI (Figure 2B) for up to 2 h of treatment. The spleen, lungs and blood were harvested 3 h after CLI.

**Figure 3:**
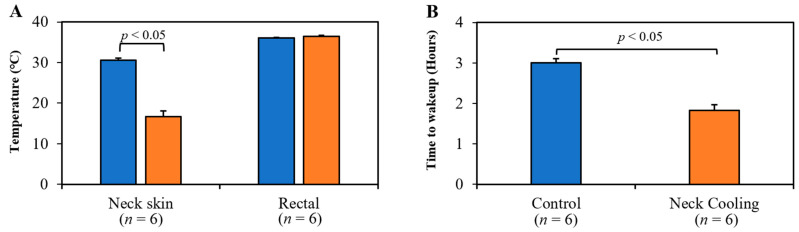
(**A**) Topical neck cooling lowered the neck skin temperature to 17 °C (*p* < 0.05 vs. control at 31 °C, left panel) without lowering rectal temperature. (**B**) Rats emerged from anesthesia significantly earlier after treatment with neck cooling.

Animal weight was comparable among all study groups. The core temperatures were maintained at normothermia throughout the experiment and were similar between control and TNC groups (36.0 ± 0.2 vs. 36.4 ± 0.3 °C, *p* = NS). Neck skin temperature was significantly lower in the TNC groups compared to the control group (16.7 ± 1.4 °C vs. 30.5 ± 0.6 °C, *p* < 0.05, Figure 3A), while maintaining normal core temperatures. Rats who underwent TNC alone emerged from anesthesia 70 min earlier than control rats (109 ± 9 min vs. 180 ± 7 min, *p* < 0.05, Figure 3B).

**Figure 4:**
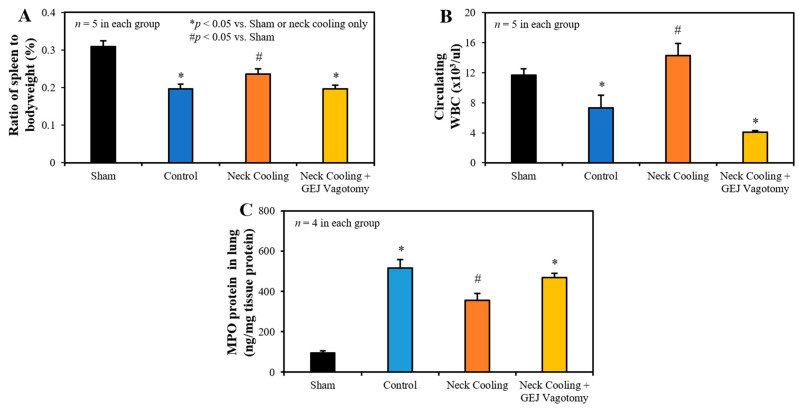
(**A**) Spleen to bodyweight ratio, (**B**) circulating white blood cells, and (**C**) myeloperoxidase (MPO) in the lung 3 h after CLI in each group. GEJ: Gastroesophageal junction.

Three hours following CLI, the control and vagotomy with TNC groups had significantly decreased splenic weight, measured as a percentage of bodyweight, and fewer circulating leukocytes than in the TNC alone group (*p* < 0.05 vs. TNC group, Figure 4A,B). In control rats, MPO, indicative of leukocyte infiltration, was significantly increased in the lung tissue. TNC significantly reduced the MPO expression in the lung compared to the control rats, but MPO levels remained significantly higher than in sham rats. Vagotomy eliminated the effect of TNC in reducing MPO in the lung (Figure 4C).

### 2.3. Survival Analysis following CLI

**Figure 5:**
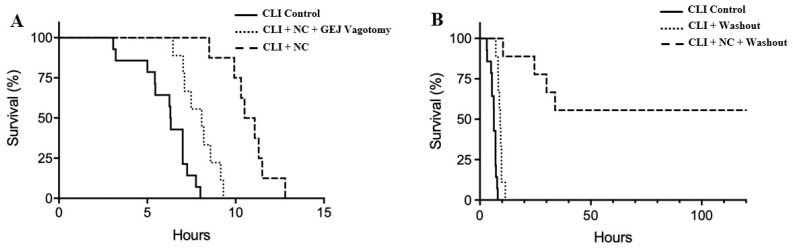
Kaplan–Meier survival curves after CLI without peritoneal washout (**A**) and with washout (**B**). NC: Neck cooling.

All control animals died within 8 h, with a median survival of 6.3 (interquartile range 5.1–7.0) h. In comparison, TNC following CLI prolonged survival to a median of 10.4 (7.7–11.4) h (*p* < 0.05 vs. control). Rats who underwent the GEJ vagotomy with TNC had a median survival of 7.9 (6.5–9.2) h, which was longer than control rats (*p* < 0.05) but shorter than rats with TNC alone (*p* < 0.05, Figure 5A).

Source control via peritoneal washout with normal saline at a dose of 0.5 mL/g weight 2 h after CLI prolonged survival duration by 3 h to a median of 9.2 (7.8–10.5) h, in comparison to the CLI control without washout (*p* < 0.05). Combining both treatments of TNC and peritoneal washout following CLI prolonged the survival duration to an average of 24 h in non-survived rats in this group (*p* < 0.05 vs. CLI + washout group). Furthermore, TNC with peritoneal washout led to a 56% survival rate (survived greater than 10 days, Figure 5B).

### 2.4. Plasma and Splenic Tissue Analysis

**Figure 6:**
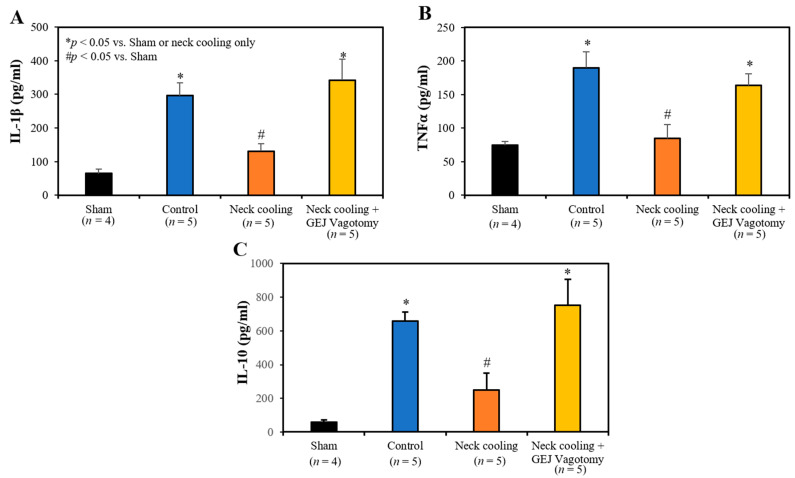
Plasma levels of (**A**) IL-1β, (**B**) TNFα and (**C**) IL-10 at 3 h after CLI.

**Figure 7:**
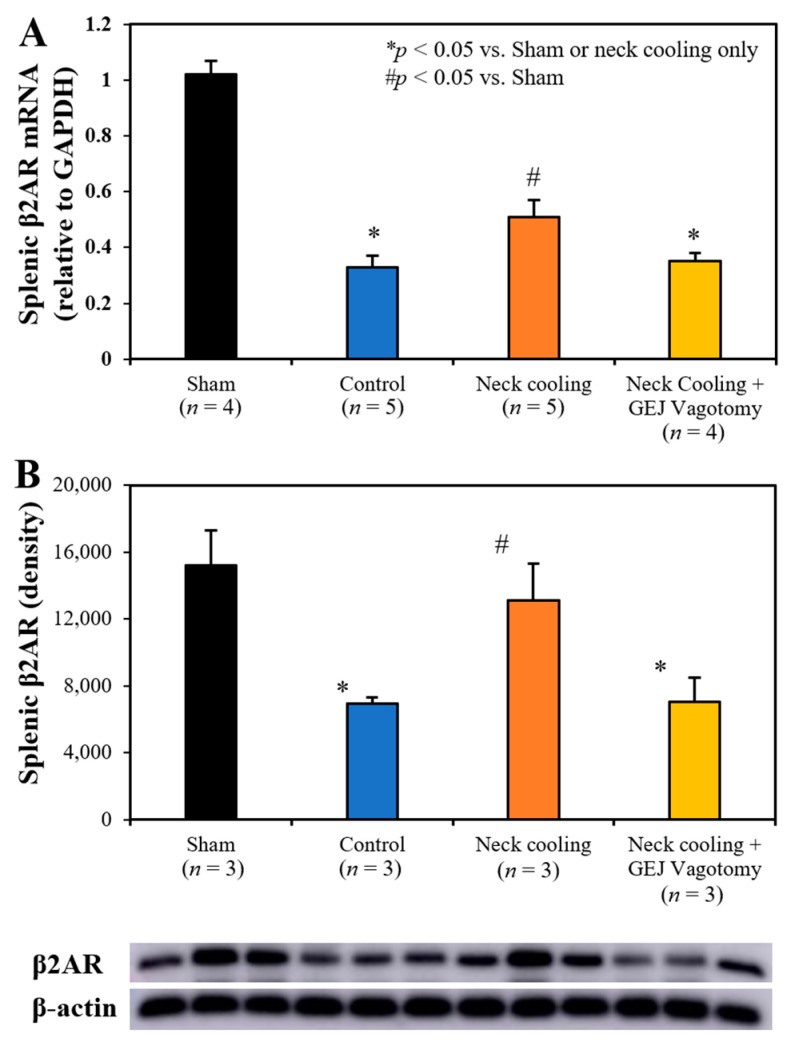
Splenic tissue expression of (**A**) β_2_-adrenergic receptor (β2AR) mRNA and (**B**) β2AR protein at 3 h after CLI.

**Figure 8:**
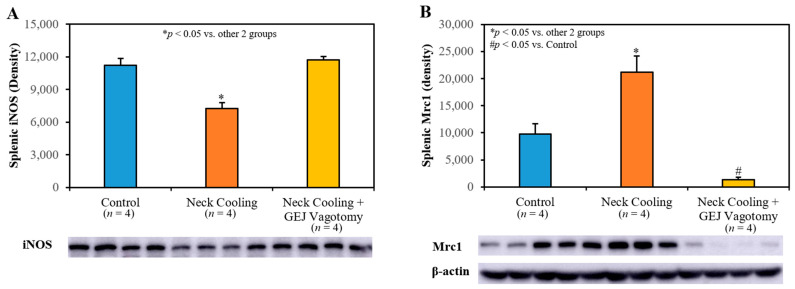
Splenic tissue expression of (**A**) iNOS and (**B**) Mrc1 protein at 3 h after CLI.

Three hours following CLI, plasma levels of IL-1β, TNF-α and IL-10 were significantly higher in the control and GEJ vagotomy groups than those in the TNC alone group (*p* < 0.05, Figure 6). Compared to sham rats without sepsis, all post-CLI groups had significantly lower β_2_-adrenergic receptor (β_2_AR) protein and mRNA levels in the splenic tissue. However, rats with TNC alone had significantly higher β_2_AR protein and mRNA levels in the splenic tissue than those in the control and vagotomy groups (*p* < 0.05, Figure 7). In the control and vagotomy with TNC groups, the expression of iNOS protein was increased but the expression of Mrc1 was decreased. These changes were reversed by TNC alone (Figure 8).

## 3. Discussion

Using a rat model with severe intraabdominal sepsis, we found that without lowering core body temperature, TNC significantly prolonged rat survival duration, similar to previous results seen with systemic hypothermia [14]. Long-term survival was achieved when a definitive source control procedure was performed following TNC treatment. TNC significantly attenuated proinflammatory responses, likely by activating a vagal anti-inflammatory pathway. Furthermore, TNC significantly shortened the recovery time from anesthesia, indicating improved tissue perfusion in these septic rats.

Sepsis can quickly progress into septic shock and multiple organ dysfunction, after which mortality rates can approach 30–40% [28,29,30]. The progression from the initial infection-related inflammatory response to acute end organ dysfunction in severe sepsis involves many pathogenic changes. Of these, the host immune system has been implicated as one of the driving forces behind the development of sepsis. Uncontrolled, excessive release of pro-inflammatory cytokines/chemokines and other mediators leads to an overt systemic inflammatory response. This inflammatory response causes alterations in microvascular flow, endothelial permeability, coagulopathy, microbiota translocation, and compromised parenchymal cell function, all of which ultimately manifest as tissue injury from inadequate perfusion and subsequent organ failure [31,32]. Mortality from sepsis is decreasing, in a large part due to evidence-based practice campaigns that have resulted in impressive improvements in outcome. However, mortality remains unacceptably high, and decades of clinical trials have yet to produce a disease-specific therapy [29,32]. Over many decades, there has been an ongoing search for new therapeutic agents to inhibit the pathogenic immunological processes that occur during sepsis. However, no new therapies have been widely incorporated into standard treatment practices [33]. Other than antibiotics, there are no approved agents on the market that are aimed at treating sepsis, and no immunomodulatory therapies have been demonstrated to be beneficial. Intraabdominal infection is one of the leading causes of sepsis, second only to pulmonary infection [4,34]. For septic patients eligible for surgical intervention, timely surgery to achieve source control of the infection is the key to preventing progression to multi-organ failure [3,4]. However, therapies to prolong this treatment window are lacking.

Using a rat model with CLI-induced severe intraabdominal sepsis, we first tested our hypothesis that TNC would prolong the survival duration. We found that TNC did significantly prolong the survival duration (Figure 5A), similar to global hypothermia as reported previously [17]. However, it did not produce an overall survival benefit. This result is plausible given that there was no source control of the intraabdominal infection with TNC alone. Nonetheless, this prolonged survival duration by TNC extended the treatment window for source control procedures. By performing peritoneal washout alone as a source control of the infection, survival duration in CLI control rats increased by three additional hours (*p* < 0.05). However, a significantly longer survival duration with a 56% survival rate (>10 days) was achieved when CLI rats were treated with both TNC shortly after CLI and then definitive peritoneal washout 2 h later (Figure 5B). Adding a source control procedure enhanced the benefit of TNC dramatically. While source control procedures are the key to treating intraabdominal sepsis, TNC plays a critical role in delaying and inhibiting ongoing proinflammatory responses and in prolonging the therapeutic window. Both interventions (TNC and source control) are required to improve overall survival in rats with severe intraabdominal sepsis.

Mechanistically, intraabdominal sepsis induced significant splenic contraction, denoted by a reduction in the splenic-weight to body-weight ratio (Figure 4A) and a significant reduction in circulating leukocytes (Figure 4B). It is plausible that the splenic contraction is related to the increase in circulating leukocytes that is expected to occur shortly after induction of intraabdominal sepsis. Then, the decrease in circulating leukocytes 3 h after CLI may portend the margination or infiltration of leukocytes to peripheral tissues such as the lungs (Figure 4C) and peritoneal cavity. MPO is a heme protein and abundantly expressed in neutrophils [35]. The increased expression of MPO in the lung indicated increased tissue neutrophil infiltration, which corresponded to decreased circulating leukocytes following CLI (Figure 4B). TNC significantly inhibited splenic contraction, preserving the splenic weight and limiting the margination and infiltration of circulating leukocytes into the peripheral tissues (Figure 4C). TNC also inhibited the production of pro-inflammatory cytokines (IL-1β, and TNFα) as well as anti-inflammatory cytokine IL-10 (Figure 6). In control rats, both pro- and anti-inflammatory cytokines were greatly elevated compared to sham rats, likely with increases in IL-10 occurring as a response to the increased levels of the pro-inflammatory IL-1β and TNFα. Thus, the low IL-10 levels after TNC are suspected to result from counteracting the pro-inflammatory cytokines, as opposed to a direct effect from TNC (Figure 6C). The inhibitory effect of TNC on proinflammatory cytokines is likely secondary to its effect of promoting the conversion of the monocytes towards the anti-inflammatory M2 phenotype, as characterized by decreased expression of iNOS and increased expression of Mrc1 (Figure 8). The effects of TNC on spleen weight, circulating leukocytes, cytokines, and M2 conversion were completely blocked by performing a vagotomy at the GEJ before CLI. These results suggest that TNC inhibits inflammatory responses by activating the vagal nerve.

Cold stimulus (temperature 16 to 19 °C) to the lateral neck, not the cheek or arm, has been shown to activate the vagal nerve [36]. Using an ice-filled Penrose drain, TNC brought the neck skin temperature down to 17 °C (Figure 3A). We found that TNC caused bradycardia in normal rats with a mild decrease in blood pressure and oxygen saturation (Figure 1). In rats with abdominal feculent sepsis and anesthetized with Ketamine/xylazine, TNC-treated rats emerged from anesthesia much earlier than control rats (Figure 3B), indicating that TNC improved tissue perfusion, particularly brain perfusion, likely in part by inhibiting systemic inflammatory responses. For those rats who survived greater than 10 days, there was no evidence of neck skin necrosis or other gross cutaneous injury.

Previous studies have shown that stimulation of a splenic- and/or vagus nerve-mediated anti-inflammatory reflex is protective against inflammatory response-mediated injury [14,37,38,39,40,41]. This pathway, termed the cholinergic anti-inflammatory pathway (CAP), begins when neural stimulation prompts the release of norepinephrine from the splenic nerve. Norepinephrine then binds β_2_ARs on splenic leukocytes, which stimulates the release of acetylcholine. Acetylcholine then binds to α7 nicotinic acetylcholine receptors on splenic macrophages to reduce leukocyte activation and inhibit the production of pro-inflammatory cytokines [42]. TNC inhibited the CLI-induced inflammatory response and preserved splenic β_2_AR protein and mRNA expression (Figure 7). Disruption of the vagus nerve at the GEJ eliminated the survival and anti-inflammatory benefit imparted by TNC. Thus, TNC activates a vagal CAP anti-inflammatory pathway to delay the progression of sepsis to an irreversible multi-organ failure pathway. 

Our results demonstrated that the application of TNC alone can only prolong the survival duration, not improve overall survival (Figure 5A). Definitive treatment to achieve source control of the infection is key to saving lives (Figure 5B). Nonetheless, TNC prolongs the treatment window to perform procedures to achieve definitive source control. Compared to therapeutic global hypothermia [18], TNC may avoid the global hypothermia-associated risks and is easily applicable, non-invasive, and has a small footprint. In the presence of bowel perforation or other life-threatening infections, this therapy would be a temporizing measure before surgery or other definitive interventions could be achieved.

Limitations: We presented in the current experiment a novel treatment for severe sepsis and found that TNC inhibited inflammatory response by activating the vagus nerve. We did not investigate other possible mechanisms underlying TNC’s effect, such as hormonal changes from the neck endocrine glands and CNS or C1 neuronal activation. TNC decreased the neck skin temperature to 17 °C. The dose–effect response with mild or deeper TNC needs to be determined. The migration of intraperitoneal bacteria into the bloodstream is, in theory, inhibited by TNC, but needs to be further defined. The effect of topical cooling on other body parts was not studied but may be a better control than normothermia.

In conclusion, TNC significantly prolonged the survival of rats with severe sepsis. It was associated with decreased expression of splenic and systemic pro-inflammatory mediators, an effect that was abrogated by vagotomy. It may be effective by activating the vagus nerve, preserving splenic β_2_AR expression and enhancing M2 conversion in macrophages, which ultimately leads to an overall reduced systemic inflammatory response. With these promising results, future studies will include pre-clinical studies in a severe sepsis swine model as well as clinical trials to evaluate efficacy and side effects, including the cutaneous impact of the treatment. Clinically, TNC may prolong the treatment window for definitive source control and avoid the myriad negative side effects seen with global hypothermia. TNC is a novel treatment that could be easily applied by patients themselves or bystanders immediately after the onset of intraabdominal sepsis in the pre-hospital setting as well. It may be especially beneficial to patients who require transfer to tertiary centers for definitive source control procedures. Given its ease of applicability and profound benefit, TNC is a promising new adjunct therapy for septic patients. 

## 4. Materials and Methods

This study conformed to the Guide for the Care and Use of Laboratory Animals published by the National Institutes of Health (8th Edition, revised 2011) and was conducted under protocols approved by the University of Virginia’s Institutional Animal Care and Use Committee (Protocol Number 3943-08-18, 13 March 2019). 

### 4.1. Animals

Adult Sprague Dawley rats (12–16 weeks old, 300–600 g, Charles River Laboratories International, Inc, Wilmington, MA, USA) were used in this study. The rats were housed in a controlled environment with free access to food and water. They had a minimum of 7 days after arrival in our facility to acclimate to their surroundings prior to being randomized. 

### 4.2. Cecal Ligation and Incision (CLI) Model

Severe intraabdominal sepsis was induced by a CLI model as described in our previous research [14]. Briefly, animals were anesthetized with an intraperitoneal injection of ketamine-HCL (50 mg/kg) and xylazine-HCL (10 mg/kg). A 3-cm vertical midline abdominal incision was made, through which the cecum was eviscerated. A 3-cm long blind-ending distal portion of the cecum was ligated with a 4-0 silk suture (Ethicon Inc., Cincinnati, OH, USA) and a 1.5-cm long incision was made along the cecal anti-mesenteric border. The cecum was manipulated to spill stool into the abdominal cavity using a flush of 5 mL of 37 °C sterile normal saline. The laparotomy was then closed in layers with 4-0 Prolene (Ethicon Inc., Cincinnati, OH, USA) for the muscular layer followed by Autoclip wound clips (Alimed, Dedham, MA, USA) for the skin. The abdomen was gently massaged to stimulate a diffuse peritonitis. After the procedure, a subcutaneous injection of buprenorphine (0.05 mg/kg) was administered for pain control. The animals were resuscitated with a dorsal subcutaneous injection of 37 °C normal saline (0.05 mL/g). Core body temperature was monitored using a rectal probe thermocouple thermometer (Omega Engineering Inc., Norwalk, CT, USA). A heating lamp was used to ensure rectal temperature was maintained between 36–37 °C.

### 4.3. Experimental Protocol and Survival Analysis

Rats were randomized into one of 4 groups: sham laparotomy, control with CLI only, or CLI followed by TNC with or without vagotomy at the GEJ. Vagotomy was performed immediately prior to CLI (Figure 2A). In TNC groups, the neck was wrapped with an ice-filled 1-inch Penrose drain 30 min after CLI (Figure 2B) for up to 2 h of treatment. If the rats awoke and moved prior to completion of 2 total hours of cooling, the treatment was stopped early. Five rats from each group were euthanized with an isoflurane overdose 3 h after CLI to harvest the spleen, blood and lungs. The rest of the rats were followed for survival duration.

An additional 2 groups of rats underwent CLI under isoflurane anesthesia with or without TNC 30 min after CLI. Then, both groups of rats underwent peritoneal washout with 0.9% sodium chloride at a dose of 0.5 mL/g weight 2 h after CLI. 

All the remaining rats were maintained in a controlled environment and monitored for the primary endpoint of survival duration by video-surveillance (Reolink, Hongkong, China). Rats were considered deceased when they developed severe labored breathing. They were then euthanized with an isoflurane overdose.

### 4.4. Hemodynamic Monitoring

Normal rats were anesthetized using an intraperitoneal injection of ketamine-HCL (50 mg/kg) and xylazine-HCL (10 mg/kg). Core rectal temperature was maintained between 36–37 °C using a heating lamp. Blood pressure, heart rate and oxygen saturation using a tail cuff and rat pulse oximeter (CODA Monitor, Kent Scientific Co., Torrington, CT, USA) were serially evaluated before, during and after 30 min of TNC.

### 4.5. Vagotomy at the Gastroesophageal Junction (GEJ)

Vagotomy was performed immediately prior to CLI. A vertical midline incision was made in the upper abdomen. The GEJ was exposed by retracting the left lobe of the liver cephalad and the stomach caudad. Both the anterior and posterior vagus nerve trunks along the esophagus were divided. Following vagotomy, CLI was performed through the same incision. The incision was then closed in two layers with 4-0 Prolene for the muscular layer and Autoclip wound clips for the skin.

### 4.6. Tissue and Plasma Analysis

Parallel experiments with control, TNC and GEJ vagotomy with TNC arms were performed for the acute phase study. A separate group underwent a sham laparotomy with no further intervention. Three hours following CLI, animals were re-anesthetized and underwent laparotomy to procure the spleens. Blood was also obtained at this time by puncturing the heart along the left parasternal intercostal space. Lastly, the lungs were harvested. 

Leukocyte count was measured using 5 µL of whole blood (Cellometer K2, Nexcelom Bioscience, Lawrence, MA, USA). The remainder of the blood was centrifuged at 3000 RPM for 20 min at 4 °C. Plasma was harvested and stored at −80 °C for subsequent cytokine analysis. Plasma concentrations of cytokines IL-10, IL-1β and TNFα were determined using commercially available enzyme-linked immunosorbent assay (ELISA) kits (Bio-Rad Laboratories, Hercules, CA, USA; LifeSpan Biosciences, Seattle, WA, USA). Splenic tissue protein and mRNA levels of the β_2_AR were determined by Western blot analysis (anti-β_2_AR antibody purchased from Abcam, Cambridge, MA, USA) and RT-PCR (β_2_AR mRNA probe purchased from Bio-Rad Laboratories, Hercules, CA, USA). Splenic tissue CD206/Mrc1 and iNOS protein levels were determined by Western blot (antibodies purchased from ThermoFisher, Waltham, MA, USA, and Abcam, Cambridge, MA, USA respectively). MPO protein in the lung tissue was measured using an ELISA kit according to the manufacturer’s instructions (Aviva Systems Biology, San Diego, CA, USA).

### 4.7. Statistical Analysis

Kaplan–Meier survival analysis was used to compare survival duration between groups and compared with a log rank test. Mantel–Haenszel hazard ratios were calculated for survival between experimental arms. Continuous data were presented as mean ± standard error of the mean. Comparison of groups was performed using the Wilcoxon rank sum test. Intra-group data were analyzed using a paired *t*-test. A *p* value of < 0.05 was considered significant for all statistical analyses, with preference given to the point estimate and 95% confidence intervals. Data analysis was conducted using Prism 7 (GraphPad Software Inc, La Jolla, CA, USA).

## Data Availability

The data presented in this study are available within this article itself, “Topical Neck Cooling Prolongs Survival of Rats with Intra-Abdominal Feculent Sepsis by Activation of the Vagus Nerve.”
